# Sonocatalytic hydrogen generation breaks the heart of super bacteria—a hydrogen solution to super bacteria?

**DOI:** 10.1093/nsr/nwad101

**Published:** 2023-04-18

**Authors:** Jianlin Shi

**Affiliations:** State Key Laboratory of High Performance Ceramics and Superfine Microstructure, Shanghai Institute of Ceramics, Chinese Academy of Sciences; Research Unit of Nanocatalytic Medicine in Specific Therapy for Serious Disease, Chinese Academy of Medical Sciences, China

It is quite difficult to kill super bacteria using routine anti-bacterial drugs and antibiotics. Actually, super bacteria exhibit an extraordinarily strong drug resistance, mainly due to the blockage of dense extracellular matrix (ECM) of bacterial biofilm against drug penetration, which is quite similar to the case of solid tumors [1,2]. How, then, does one effectively overcome this ECM barrier? Currently available clinical approaches are not very effective. In recent years, hydrogen molecules (H_2_) have been recognized as being able to cross various biological barriers, including the blood-brain barrier and plant barriers [3], owing to its small molecular size, which also presents a bioenergy-modulating effect capable of inducing apoptosis in various tumor cells and bacteria [4]. Therefore, H_2_ medication for anti-biofilm and anti-tumor is a promising therapeutic strategy by virtue of their super penetrability.

Recently, nanocatalytic medicine has received increasing attention [5,6]. Different from routine toxic drugs, engineered nanocatalysts for biomedical applications can specifically exhibit therapeutic function at the site of disease through catalytic reaction in response to internal/external field(s) but remain catalytically inactive in normal cells/tissues. Typically, nanocatalytically generated reactive oxygen species (ROS) can kill bacteria by oxidizing their membrane and blocking the electric transfer chain of contacted bacteria [7]. Unfortunately, the extremely short lifetime (at ms scale) and work distance (at nm scale) of ROS  makes treatment of those bacteria at the deep biofilm site not very effective.

**Figure 1.**
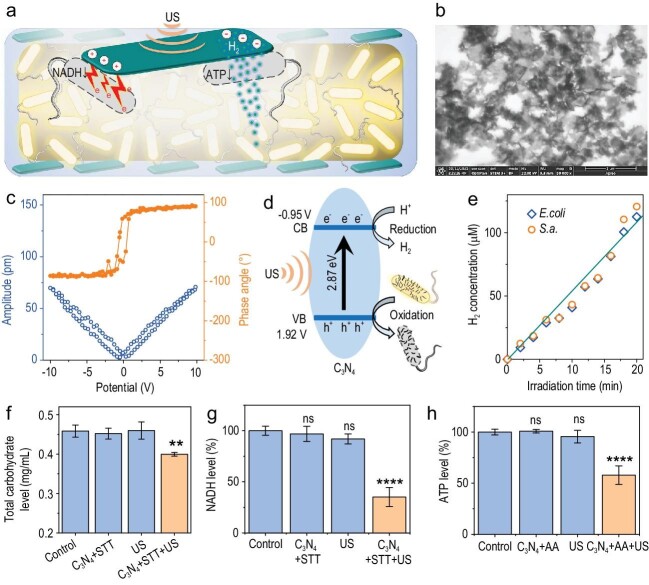
Performance and mechanism of C_3_N_4_-mediated sonocatalytic hydrogen/hole-combined anti-biofilm. (a) A schematic illustration of the sonocatalytic hydrogen/hole-combined ‘inside/outside-cooperation’ anti-biofilm mechanism. STEM image (b), the piezoelectric hysteresis loops (c), and the schematic illustration of band structure (d) of C_3_N_4_ nanosheets for sonocatalytic H_2_ production and bacterial oxidation. (e) Time-dependent sonocatalytic performance of C_3_N_4_ nanosheets for hydrogen production. Total carbohydrate levels in the *S.a.* biofilms (f), NADH levels (g) and ATP levels (h) of biofilm bacteria by different treatments. Reprinted with permission from Ref. [8].

Recently, a team led by Prof. Qianjun He has proposed a novel and interesting strategy of sonocatalytic hydrogen/hole-combined ‘inside/outside-cooperation’ anti-biofilm (Fig. 1a), which has been published most recently under the title of ‘Sonocatalytic hydrogen/hole-combined therapy for anti-biofilm and infected diabetic wound healing’ [8]. This work developed biocompatible C_3_N_4_ nanosheets as an ultrasound (US)-responsive piezoelectrocatalyst with a right band structure (Fig. 1b–d), which enables the use of polysaccharide/NADH on the surface of biofilm as a sacrificial agent for efficient sonocatalytic hydrogen generation (Fig. 1e–g) [8]. Importantly, sonocatalytically generated H_2_ can play a Trojan horse to easily penetrate into the biofilm castle for cooperating with holes outside of the biofilm. In a mouse model of biofilm-infected diabetic wound, the developed sonocatalyst shows an outstanding wound healing outcome due to the effective eradication of biofilm. It is worth noting that sonocatalytic hydrogen therapy is particularly useful for treating deep-sited diseases including deep infection and tumors thanks to an almost unlimited tissue penetration of US and H_2_ [8,9].

Furthermore, they have found that H_2_ can kill the bacteria inside biofilm by inhibiting their respiration (Fig. 1h), which is quite similar with its tumor-inhibiting effect [9]. Significantly, they have also uncovered that Fe-porphyrin is a primary molecular target/biosensor of H_2_, which is able to catalyze the CO_2_ reduction into CO by H_2_ in a hypoxic microenvironment for mediating downstream signaling [10]. It is well known that the interiors of both big solid tumors and biofilm are highly hypoxic, and therefore it is believed that the above bacterial respiration-inhibiting effect of H_2_ could derive from heme in hypoxic bacteria, which, of course, needs further experimental evidence. Nevertheless, their discovery opens a new window for major disease treatments by hydrogen medicine.
